# In Vitro Activities of Ertapenem and Imipenem against Clinical Extended Spectrum Beta-Lactamase-Producing Enterobacteriaceae Collected in Military Teaching Hospital Mohammed V of Rabat

**DOI:** 10.1155/2012/646480

**Published:** 2012-06-27

**Authors:** M. Elouennass, A. Zohoun, A. El Ameri, N. Alem, J. Kasouati, Y. Benlahlou, I. El Yaagoubi, M. Frikh, A. Lemnouer, A. Benouda

**Affiliations:** ^1^Department of Bacteriology, Military Teaching Hospital Mohammed V and Faculty of Medicine and Pharmacy, Mohammed V University, Souissi, Rabat, Morocco; ^2^Laboratory of Biostatistics, Faculty of Medicine and Pharmacy, Mohammed V University, Souissi, Rabat, Morocco; ^3^Study Research Group for Antibiotic Resistance and Bacterial Infections, Faculty of Medicine and Pharmacy, Mohammed V University, Souissi, Rabat, Morocco

## Abstract

*Objective*. To study the sensitivity level of extended spectrum beta-lactamase-producing Enterobacteriaceae to Carbapenems (Imipenem, Ertapenem) marketed in Morocco and discusses the place of Ertapenem in the treatment of extended spectrum-beta-lactamase-producing. *Materials and Methods*. A retrospective study of 110 extended spectrum beta-lactamase-producing Enterobacteriaceae. Isolates obtained from blood cultures, superficial and deep pus, and catheters were conducted. The minimum inhibitory concentrations of Imipenem and Ertapenem were done by the *E*-test. The modified Hodge test was conducted for resistant or intermediate strains. *Results*. 99.1% of isolates were susceptible to Imipenem. For Ertapenem, 4 were resistant and 4 intermediate. The modified Hodge test was positive for all 08 isolates. A minimum inhibitory concentration comparison of *K. pneumoniae, E. cloacae,* and *E. coli* for Imipenem has noted a significant difference between *E. cloacae* on one hand and *E. coli, K. pneumoniae* on the other hand (*P* < 0.01). No significant difference was noted for minimum inhibitory concentration of Ertapenem. *Conclusion*. Our results confirm in vitro effectiveness of Ertapenem against extended spectrum beta-lactamase-producing Enterobacteriaceae as reported elsewhere. However, the emergence of resistance to Carbapenems revealed by production of carbapenemases in this study confirmed a necessary bacteriological documented infection before using Ertapenem.

## 1. Introduction

Extended spectrum beta-lactamase-producing Enterobacteriaceae (ESBL-EB) represent a major health problem because of their multiple resistances to antibiotics. Treatment options are limited, often using the Carbapenems, cephamycins, fosfomycin, furans, and colimycin [1–4]. The results of clinical studies suggest that Imipenem remains the primary choice of treatment for bacteria that produces ESBLs [5–9]. These results increase in overall the prescription of Imipenem, an overbill and an additional selection pressure on the ecosystem, causing and maintaining in our region the multidrug resistance *Acinetobacter baumannii* and *Pseudomonas aeruginosa* endemicity, and recently the emergence of Enterobacteriaceae carbapenem-resistant strains [10, 11]. In Morocco, there are two available Carbapenems: Imipenem (IMP) and Ertapenem (ERT). Ertapenem is the second molecule of the family on the market since 2008. 

The aim of our work was to study the ESBL-EB sensitivity to Carbapenems marketed in Morocco, to discuss the impact of use of Imipenem on the emergence of resistance to Carbapenems, and the Ertapenem place's in the ESBL-EB treatment.

## 2. Materials and Methods

A retrospective study was conducted between January 2009 and September 2010 in the Department of Bacteriology of the Military Teaching Hospital Mohammed V of Rabat (HMIMV). Isolates of Enterobacteriaceae with a resistance phenotype-type ESBLs from blood cultures, samples of superficial and deep pus, and catheters were included. Isolates of Enterobacteriaceae without ESBL phenotype and/or isolated from the urogenital and lung samples were excluded. Duplicates were also eliminated. 

Identification of Enterobacteriaceae isolates was performed by the API 20E (BioMerieux, Marcy l'Etoile, France). The detection of ESBL phenotype was performed as recommended by the Antibiogram Committee of the Microbiology French Society (CASFM) [12]. The minimum inhibitory concentrations (MIC) of IMP and ERT were determined by the *E*-test according to the manufacturer's recommendations and interpreted as recommended by the CASFM (ERT: *S* ≤ 0.5;  *R* > 1 ; IMP  *S* ≤ 2;  *R* > 8). The modified Hodge test was performed for resistant strains and/or intermediate to IMP and/or ERT using the technique described by Lee et al. [13]. Quality control was performed with an *Escherichia coli* local wild strain identified in house. Statistical analysis was performed using the SPSS 13.0 software and the results expressed as percentages for qualitative variables and as mean ± standard deviation or median and quartiles for quantitative variables. The comparison between the MIC of the different species was performed by the Kruskal-Wallis test. 

## 3. Results

During the study period, 384 EB were isolated of which 110 (28.6%) had an ESBL phenotype. From blood cultures, 103 EB were isolated of which 43 (41.7%) had an ESBL phenotype. From samples of pus, 239 EB were isolated of which 43 (18%) hadanESBLphenotype. From sampling catheters, 42 EB were isolated of which 24 (57.14%) hadanESBLphenotype. The distribution of ESBL-EB by species, type of collection, and service is illustrated in Table 1.

Susceptibility to Carbapenems of ESBL-EB isolates was 99.1% for IMP (109 susceptible and one intermediate: *E. cloacae* with a MIC of 3 *μ*g/mL). For ERT, 102 isolates were sensitive (92.8%), 4 intermediate (3.6%), and 4 resistant (3.6%) whose 2 *K. pneumoniae*, one *E. coli* and one **E. * cloacae*. The strain of *E. cloacae* resistant to Ertapenem is the same which is intermediate to IMP. The modified Hodge test was positive for 08 of intermediate and resistant isolates to ERT. The IMP's and ERT's MIC distributions are shown in Table 2. 

The MIC results were expressed as median and quartile since their distribution does not follow a normal distribution: IMP: 0.19 *μ*g/mL [0.125, 0.25]; ERT: 0.125 *μ*g/mL [0.032, 0.25]. Distributions of MICs of IMP and ERT of 03 major species of ESBL-EB are represented, respectively, in Tables 3 and 4. 

Comparison of MIC of the three major species (*K. pneumoniae*, *E. cloacae*, and *E. coli*) has noted for IMP, a statistically significant difference between *E. cloacae* one hand and *E. coli* and *K. pneumoniae* on the other  (*P* < 0.01). No statistically significant difference was noted for three major species with respect to the MIC of ERT.

## 4. Discussion

The high prevalence of ESBL-EB, particularly in blood cultures (10% of positive blood cultures and 41.7% of EB isolated) in our hospital, is a major health problem. It concerns more *K. pneumoniae* than *E. cloacae* and *E. coli * but the* E. cloacae *infection could be more difficult to treat, because of intrinsic AmpC production. The multiresistance of ESBL-EB limitsthe use of antibiotics to onlyImipenem and secondarily to Ertapenem, piperacillin/tazobactam, fosfomycin, and colistin [5, 14]. In fact, in vivo and in vitro data confirms that Imipenem is the best treatment for ESBL-EB infections [6–9]. In our study, in vitro activity of the two Carbapenems marketed in Morocco was determined using *E*-test. Susceptibility to Imipenem was 99.1%, only one isolate was intermediate. By cons, sensitivity to Ertapenem was 92.8% with four resistant (3.6%) and four intermediate (3.6%) isolates. All intermediate or resistant isolates had a positive Hodge test, demonstrating the production of carbapenemases but the typing was not performed. Considering the very limited use of Ertapenem in our establishment the resistance rate to 7.2% is maybe the base rate of resistance to Ertapenem. The median of IMP MIC was 0.19 *μ*g/mL (0.125, 0.25) and the median of ERT MIC was 0.125 *μ*g/mL (0.032, 0.25), so 75% of MIC's are ≤0.25 *μ*g/mL for both molecules. The in vitro activity of Ertapenem against the ESBL-EB was less than that of Imipenem. The rates of MIC of *E. cloacae* isolates are higher than those of *K. pneumoniae* and *E. coli* with a statistically significant difference (*P* < 0.01). In effect, 24% of *E. cloacae* isolates had an MIC (ERT) range of 0.75 to 1.5, 85% MIC (IMP) <0.5 *μ*g/mL and 71% MIC (ERT) <0.25 *μ*g/mL. The cephalosporinases hyperproduction associated with ESBL maybe explains the increase of MIC without a production of carbapenemases. These results confirm, the literature data which indicates that Ertapenem is active against ESBL-EB like *E*. *coli* and *Klebsiella,* but the activity is more limited for other ESBL-EB like *Enterobacter spp*. However, in the presence of ESBL or high produced cephalosporinases, there is usually an increase of two to eight times in the MIC of Ertapenem [15, 16]. Despite this efficiency in vitro, the use of Ertapenem as an alternative, suffers from the poverty of clinical data with often retrospective studies [17–20]. Furthermore, this use of Ertapenem, in our region, should consider two basic elements: first, the emergence of carbapenemases producing strains revealed by our study and reported by Benouda et al. [11]. This emergence is associated, in some publications, to treatment with Ertapenem [21, 22]. Second, the prescription of Carbapenems would generate a selective pressure on bacterial ecosystem and would participate in the *P. aeruginosa* and *A. baumannii resistant* Imipenem or pan-resistant strains endemicity, and consequently, this increases the risk of reducing the antibiotic arsenal. 

The question arises whether the introduction of Ertapenem will have a reducing effect on the Imipenem resistance rates of *P. aeruginosa *and *A. baumannii,* species naturally resistant to Ertapenem. Some authors report that, the use of Ertapenem may help to improve the Imipenem sensitivity of *P. aeruginosa* by reducing unnecessary use of the IMP and the reduction of selection pressure [23–27]. According to Livemore et al., the wise use and consistency with the recommendations of the marketing authorization does not cause high risk or an additional selection of mutant's resistants (*P. aeruginosa, A. baumannii)* to Carbapenems including Imipenem [28]. A study has concluded has concluded that there was no association between the changes in the sensitivity of *P. aeruginosa* to Carbapenems in 25 hospitals after 9 years of using Ertapenem [29]. In light of these data, Ertapenem should be usedonly in hospitals, preferably after bacteriological documentation or first line as required by the marketing authorization, after amedico-economic evaluationand ifthe bacterial ecology showsa significant resistancerate of Enterobacteriaceaeor if noalternative for ESBL-EB suspected infection. A reassessment is mandatory after bacteriological documentation and therapeutic de-escalation, if possible. The treatment duration should be as short as possible and dosage sufficient especially in the early phase of infection (high inoculum) [19, 20, 30]. A surveillance policy and prevention are necessary to control the emergence of multiresistant strains. This justifies the efforts to prevent the spread of carbapenemases producing strains, including strict compliance with the antibiotic treatment strategies recommendations in general and in particular the use of Carbapenems [31, 32].

## 5. Conclusion

Although Carbapenems available in our area (Imipenem and Ertapenem) have a good activity on extended spectrum beta-lactamase-producing Enterobacteriaceae, our study reveals the existence of strains producing carbapenemases resistant to Ertapenem. This encourages the wise use of Ertapenem as an alternative to Imipenem in specific situations and efforts to prevent the emergence of these strains and their dissemination.

## Figures and Tables

**Table 1:** Species and samples distribution of ESBL-EB (*N* = 110).

Species (no. of isolates tested)	No. (%) of isolates with ESBL			
	Blood	Pus	Catheter	Total
*K. pneumoniae* (94)	27 (39.7)	21 (30.9)	20 (29.4)	**68 (17.7)**
*E. cloacae* (67)	9 (40.9)	9 (40.9)	4 (18.2)	**22 **(5.7)
*E. coli* (98)	6 (33.3)	12 (66.7)	0	**18 (4.7) **
*P. mirabilis* (49)	0	1 (100)	0	**1 **(0.03)
*P. stuartii* (4)	1 (100)	0	0	**1 (0.03)**

Total **(384)**	**43 **(39.1)	**43 **(39.1)	**24 **(21.8)	**110 **(28.6)

**Table 2:** Imipenem (IMP) and Ertapenem (ERT) MIC distribution of extended spectrum beta-lactamase-producing Enterobacteriaceae.

% of Isolates		
MIC'S	IMP	ERT
**0.012**	0.9	3.7
**0.016**	0	5.6
**0.023**	0	9.3
**0.032**	0	7.4
**0.038**	0.9	0
**0.047**	0	5.5
**0.064**	0.9	4.6
**0.094**	7.5	6.4
**0.125**	22.4	14.8
**0.19**	39.2	10.2
**0.25**	13.1	10.2
**0.38**	2.8	9.3
**0.5**	5.6	5.5
**0.75**	1.8	3.7
**1**	0.9	0
**1.5**	1.8	0.9
**2**	0.9	0.9
**3**	0.9	0
**8**	0	0.9
**32**	0	0.9

**Table 3:** Imipenem (IMP) MICs distribution of 03 principals species of ESBL-EB.

% of isolates			
IMP MIC	*K. pneumoniae *	*E. cloacae *	* E. coli *
**0.012**	1.5	0	0
**0.032**	0	4.8	0
**0.064**	1.5	0	0
**0.094**	7.6	4.8	11.1
**0.125**	27.3	4.8	27.8
**0.19**	45.4	14.3	50
**0.25**	10.6	23.8	11.1
**0.38**	0	9.5	0
**0.5**	1.5	23.8	0
**0.75**	3	0	0
**1**	1.5	0	0
**1.5**	0	4.8	0
**2**	0	4.8	0
**3**	0	4.8	0

**Table 4:** Ertapenem (ERT) MICs distribution of 03 main species of ESBL-EB.

% of isolates			
ERT MIC	* K. pneumoniae *	*E. cloacae *	*E. coli *
**0.012**	6	0	0
**0.016**	7.5	0	4
**0.023**	10.4	0	0
**0.032**	10.4	4,8	0
**0.047**	3	0	9.5
**0.064**	6	0	0
**0.08**	0	0	0
**0.094**	3	4.8	9.5
**0.125**	14.9	4.8	9.5
**0.19**	7.5	14.3	23.8
**0.25**	10.4	23.8	14.3
**0.38**	9	9.5	0
**0.5**	8.9	23.8	0
**0.75**	0	0	19
**1.5**	0	4.8	4.8
**2**	0	4.8	4.8
**3**	0	4.8	0
**8**	1.5	0	0
**32**	1.5	0	0
